# Krüppel-like Factor 15: A Potential Therapeutic Target For Kidney Disease

**DOI:** 10.7150/ijbs.34838

**Published:** 2019-07-21

**Authors:** Lefeng Wang, Weiqiang Lin, Jianghua Chen

**Affiliations:** 1 The Kidney Disease Center, The First Affiliated Hospital, Zhejiang University School of Medicine, Hangzhou 310003, China.; 2 Institute of Translational Medicine, Zhejiang University School of Medicine, Hangzhou 310029, China.

**Keywords:** Krüppel-like factor 15, Kidney disease, Podocyte differentiation, Mesangial cell, Renal fibrosis

## Abstract

Krüppel-like factor 15 (KLF15) is a zinc-finger transcription factor highly expressed in the glomeruli and interstitial cells of kidneys. An increasing number of studies have demonstrated a critical role for KLF15 in the kidney, involving tubular physiology, podocyte injury, renal fibrosis, and mesangial pathology. In this review, we discuss recent advances and update our overview of the functions of KLF15 in kidney biology, hoping to provide new perspectives on the progression and therapy of Chronic Kidney Disease (CKD). A better understanding of KLF15 with respect to its diverse roles in specific cells or diseases will be beneficial in pursuing novel therapeutic targets and moving forward to precision medicine.

## Introduction

Chronic Kidney Disease (CKD) is highly prevalent in many countries 1-4, increasing the risk of cardiovascular disease, cognitive dysfunction, as well as general mortality 4, 5. Individuals with CKD may gradually develop end-stage renal disease (ESRD) and need to receive renal replacement therapy (RRT) 3, 6, 7. RRT is available in developed countries but difficult to access in poor areas 8, 9, and greatly affects the quality of life of patients. Therefore, CKD has become a global public health problem, and efficient therapeutic targets are urgently needed to slow CKD progression and improve prognoses 10, 11.

Krüppel-like factors (KLFs) are a group of zinc-finger DNA-binding transcription factors involved in various biological processes, such as cell differentiation, metabolism, inflammation, apoptosis, mitochondrial biogenesis, DNA repair, and many others 12-15. The KLFs family consist of eighteen members, among which KLF15, is widely distributed in the glomeruli and interstitium of kidneys, pancreas, heart, liver, and skeletal muscles 16. Previous studies have demonstrated that KLF15 is a key transcriptional regulator in diverse physiological processes, including gluconeogenesis, immune response and adipocyte differentiation 17-20. Moreover, KLF15 was also involved in metabolic dysfunctions, including diabetes, inflammation, obesity, and cardiac hypertrophy 17-20. Recently, a growing number of studies have indicated that KLF15 is implicated in renal physiological processes and pathologic progression of CKD, involving podocyte differentiation, tubular physiology, mesangial pathology, and renal fibrosis 12, 13, 16, 21. An improved understanding of CKD and its molecular mechanisms will enable further research into new therapeutic targets.

Herein, we summarize relevant studies and highlight recent updates on KLF15 and its role in renal biology, hoping to provide insights into potential areas for further investigation.

## Structure and function of KLF15

Human KLF15 is mapped at 3q21.3 according to human chromosome mapping 22. The KLF15 cDNA is composed of a single open reading frame of 1248 bp, encoding a polypeptide of 416 amino acids 22, 23. Like other members of the KLFs family, KLF15 protein contains a conserved three-zinc-finger (C2H2) motif in the C-terminal region, which can bind to GC-rich sequences in target gene promoters and regulate transcription levels 24. Though the DNA-binding ability is similar, KLFs family have distinct transactivation or transrepression domains in their N-terminal region, leading to different tissue distribution patterns and biological functions of KLFs. According to the differences, KLFs can be divided into three groups 25. Members of the first group act as transcriptional repressors by interacting with C-terminal binding protein (CtBP) 25. KLFs in the second group work as transcriptional activators 25, and members of the third group serve as transcriptional repressors through an α-helical motif, which can mediate their binding to Sin3A 25. However, KLF15 is not a member of any of these three groups, because the protein interaction domains of KLF15 have not been determined yet. Thus, a more specific illustration of its structure requires further research.

## Roles of KLF15 in kidney physiology

### KLF15 in renal tubular physiology

KLF15 was first described as Kidney-enriched Krüppel-like factor (KKLF), a gene found to be expressed in human mesangial cells, interstitium, and the nephron segment where the kidney-specific chloride channels CLC-K1 and CLC-K2 were absent 22. In rat kidney cells, KLF15 appeared to suppress the expression of CLC-K1 and CLC-K2 in the thin descending limb of Henle's loop and inner medullary collecting ducts by competing for promoter binding with myc-associated zinc-finger (MAZ) protein, a transcription factor able to activate CLC-K1 and CLC-K2 22, 23. This finding indicates that KLF15 is associated with the nephron segment-specific expression of CLC-K1 and CLC-K2 genes 23. However, it still remains unclear whether KLF15 is functional in chloride transport 12.

### KLF15 in podocyte differentiation

**Figure 1:**
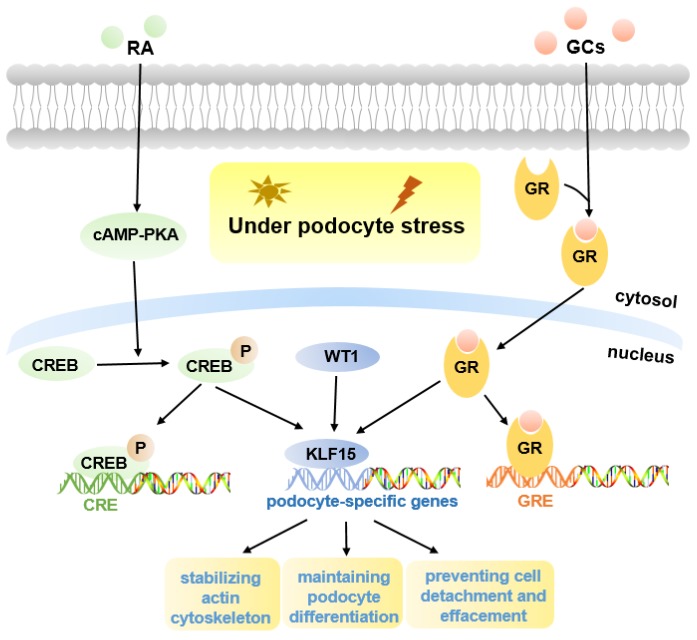
** Molecular mechanisms of KLF15 in podocyte injury.** KLF15 mediates RA and GCs-induced restoration of podocyte differentiation markers (Nephrin, Synaptopodin, and Podocin) under podocyte stress. Pathways activated by KLF15 include stabilization of the actin cytoskeleton, podocyte differentiation, and focal adhesion. The activity of KLF15 is partly mediated by WT1.

Podocyte is highly differentiated in normal mature kidneys 12. The loss of podocyte differentiation markers is regarded as a main manifestation of podocyte injury 26. Previous studies have shown that retinoic acid (RA) may induce podocyte differentiation via activation of the cAMP pathway, however, there appear to be other pathways, as binding sites for cAMP-response elements (CREB) are lacking in promoters of many podocyte maturity marker genes 27. One hypothesis is that podocyte differentiation may be mediated by RA-induced transcription factors, and microarray gene expression studies in human podocytes identified a differentially regulated gene, *KLF15*, which was up-regulated in both murine and human podocytes treated with RA 27, 28. Subsequently, KLF15 was shown to regulate RA-induced restoration of podocyte maturity markers by transcriptionally activating podocyte-specific genes (*Synaptopodin*, *Podocin*, and *Nephrin*) under cell stress 27 (Fig. 1).

### The functions of KLF15 in *Drosophila* nephrocytes

Interestingly, a study demonstrated a vital role of KLF15 in the development and differentiation of the *Drosophila* nephrocyte, a research model of human podocytes 29. The nephrocyte of *Drosophila* is a highly endocytic cell, which expresses genes that are evolutionarily conserved in human podocytes 30. In the *Klf15* loss-of-function mutants, nephrocyte development was impaired during embryogenesis. In addition, a loss of endocytic scavenger function was also observed in the adult *Drosophila* nephrocytes when *Klf15* was conditionally silenced 29. Inversely, overexpression of *Klf15* led to the proliferation of nephrocytes and the improvement of cell function during ageing process 29. Additionally, *Klf15* was involved in a nephrocyte-restricted differentiation pathway, indicating that *Klf15* was essential in maintaining the differentiation of *Drosophila* nephrocytes 29. Future research may use *Drosophila* to explore further functions and mechanisms of KLF15 in human podocytes.

## Roles of KLF15 in kidney disease

### KLF15 in podocyte injury

Pathological mechanisms of CKD include cellular injury, inflammation, podocyte effacement, proteinuria, and glomerulosclerosis 31. Podocyte injury is characterized by loss of mature podocyte differentiation markers and destabilization of the actin cytoskeleton in response to excessive stress. It is regarded as the hallmark of various glomerular diseases, such as Focal Segmental Glomerulosclerosis (FSGS), Membranous Nephropathy (MN), Minimal Change Disease (MCD), and HIV-Associated Nephropathy (HIVAN) 27, 32, 33.

Previous studies indicated that podocyte-specific *Klf15*-KO mice in the unperturbed state did not develop significant proteinuria or glomerulosclerosis, but in Lipopolysaccharide (LPS) or Adriamycin (ADR)-induced proteinuric murine models, loss of KLF15 led to a significant increase in the susceptibility to podocyte injury, indicating a critical role for KLF15 as a regulator of podocyte differentiation protecting against podocyte injury 27 (Fig. 1). Similarly, a lower expression of KLF15 in podocytes from patients with primary glomerulopathies, including FSGS and HIVAN, also confirmed this view 27; a lower expression seems to be correlated with worse renal outcomes for patients 34.

Glucocorticoids (GCs) are usually the primary therapy for glomerular diseases 35. Recent studies demonstrated that, in both murine and human podocytes treated with GCs, the KLF15 levels were dramatically increased, and the affinity of *KLF15* promoters binding with GCs-receptor was also improved 16. Loss of KLF15 in murine podocytes weakened the beneficial effect of GCs, resulting in destabilization of the actin cytoskeleton and increased podocyte injury 16. In contrast, overexpression of KLF15 under podocyte stress led to improved stabilization of the actin cytoskeleton 16. Moreover, KLF15 levels in the glomeruli of patients with glomerular diseases were associated with the responsiveness to GCs 16. Consequently, these studies identified important effects of KLF15 in mediating the benefits of GCs in podocytes (Fig. 1).

Excitingly, a recent report showed that podocyte-specific induction of *Klf15* in ADR-induced and HIV-1 transgenic (Tg26) proteinuric murine models, can attenuate renal fibrosis, inflammation and podocyte injury, contributing to improved cell survival 13. Enrichment analyses have indicated that induction of *Klf15* is implicated in the restoration of podocyte differentiation markers, stabilization of the actin cytoskeleton, as well as focal adhesion. Therefore, KLF15 may be a novel therapeutic target in proteinuric diseases 13. Moreover, Wilms Tumor 1 (WT1) was observed to regulate the activity of KLF15 in part (Fig. 1). WT1 is a critical podocyte-specific transcription factor, and plays an essential role in kidney development, indicating the potential functions of KLF15 in kidney development 13.

### KLF15 in mesangial pathology

High glucose level is a critical contributor to diabetic nephropathy; *in vitro* evidence indicates that it can promote the proliferation of mesangial cells 36. KLF15 can inhibit the high glucose-induced cell proliferation, and repress the development of diabetic nephropathy 36. Possible mechanisms are associated with the extracellular-regulated kinase (ERK)/MAPK signaling pathways 36. Therefore, KLF15 may work in the therapy of diabetic nephropathy.

**Figure 2:**
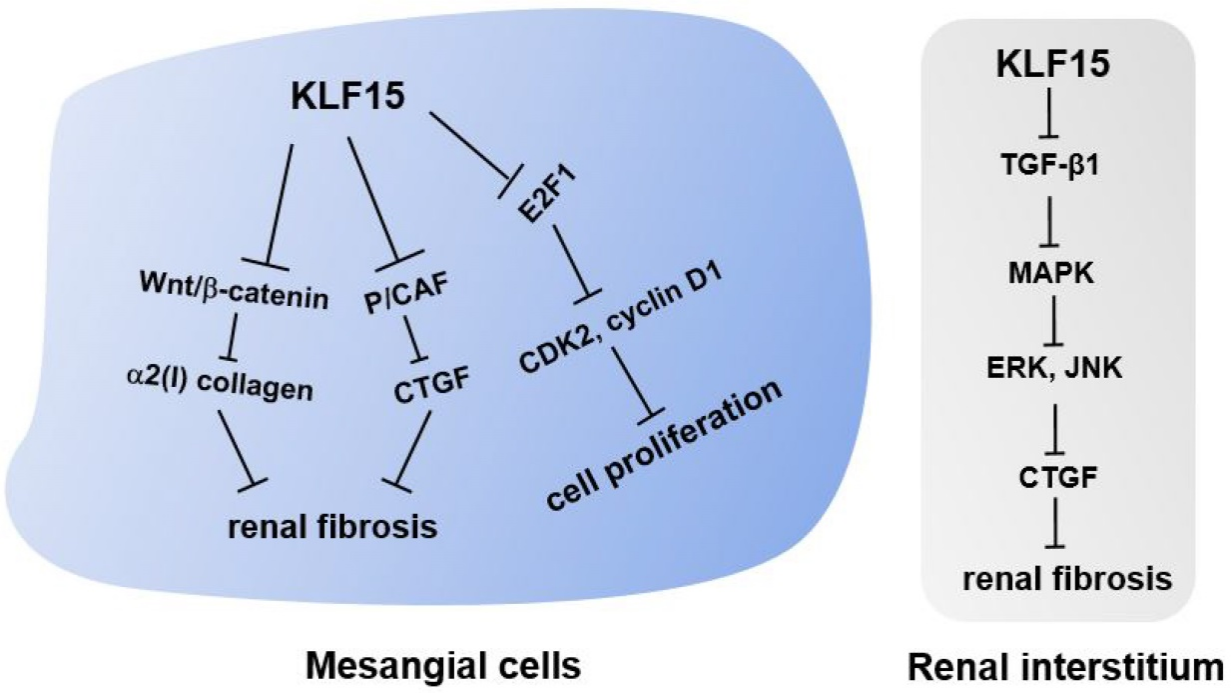
** Molecular mechanisms of KLF15 in renal fibrosis and mesangial pathology.** KLF15 plays an antifibrotic role in renal fibrosis through inhibiting the canonical Wnt/β-catenin pathway and suppressing the recruitment of P/CAF to the *CTGF* promoter in mesangial cells. In the renal interstitium, KLF15 attenuates the deposition of extracellular matrix and CTGF by inhibiting TGF-β1-mediated JNK/MAPK and ERK/MAPK signaling pathways. In addition, KLF15 expression in mesangial cells inhibits the proliferation of mesangial cells via repressing the cell cycle regulation factor E2F1.

In a classic anti-Thy1 mesangial proliferative nephritis rat model, KLF15 expression in mesangial cells inhibited cell proliferation by down-regulating the expression of the cell cycle regulation factor E2F1, a downstream target of KLF15 37 (Fig. 2). Thus, KLF15 is expected to be a potential therapeutic target for mesangial proliferative glomerulonephritis, a common non-specific change characterized by immune complex deposition, podocytopathies, and complement alterations 37.

### KLF15 in Lupus Nephritis

Lupus nephritis (LN) is known to cause permanent renal injury. The issue of treatment-related toxicity in LN remains to be resolved, which has promoted the development of drug combinations 39-41. Previous studies have shown that KLF15 can block the interaction of nuclear factor NF-κB p56 with the co-activator P300 by directly interacting with P300, and subsequently by inhibiting the P300-p56-activated inflammatory pathway 42. The most recent evidence has indicated that KLF15 can improve LN 21. A combined treatment of Hydroxychloroquine and artemisinin in LN may exert renal-protective effects by increasing expression levels of KLF15, leading to a decrease in the levels of its downstream target, NF-κB, and consequently suppressing the inflammatory response 21.

### KLF15 in renal fibrosis

Renal fibrosis, a dynamic process in response to excessive epithelial injury, consists of glomerulosclerosis and tubulointerstitial fibrosis due to extracellular matrix accumulation 43. Renal fibrosis is regarded as the histological manifestation and hallmark of progressive CKD, as well as a reliable indicator of prognosis 7, 44. Multiple factors are associated with the progression of fibrosis, such as oxidative stress, inflammation, and transforming growth factor-β (TGF-β) 45. Herein, we provide an update on the research literature regarding the role of KLF15 on renal fibrosis (Fig. 2).

When first described in the kidney, KLF15 was suggested to play a role in fibrogenesis, given its localization in potentially fibrogenic mesangial cells and tubulointerstitial compartments 16, 22. This notion was verified when authors observed a decreased expression level of KLF15 followed by increased levels of α2(I) collagen in kidneys of unilateral ureteral obstruction (UUO) mice, a classic murine model of progressive renal fibrosis 22, 46. Subsequent experiments demonstrated that KLF15 repressed the activity of α2(I) collagen promoter, thus inhibiting the synthesis of type I collagen 22, 23. Besides, knockdown of *Klf15* in Foxd1^+^ stroma cells exacerbated the proliferation of myofibroblasts and the deposition of extracellular matrix in UUO and angiotensin II-treated mice, two murine models of renal fibrosis 46. Combined with *in vitro* experiments, these results confirmed that KLF15 was critical in attenuating renal fibrosis by inhibiting the canonical Wnt/β-catenin pathway 46. In the angiotensin II-treated murine model, KLF15 was likely to inhibit the expression of angiotensin II-induced profibrotic connective tissue growth factor (CTGF) by suppressing the recruitment of the co-activator p300/CREB-binding protein-associated factor (P/CAF) to the *CTGF* promoter, thus exerting an early antifibrotic effect in renal fibrosis 18.

Interestingly, another report demonstrated that a low-protein diet increased renal KLF15 expression levels in normal and 5/6-nephrectomized rats, a remnant kidney model of progressive renal fibrosis 45. KLF15 levels in mesangial cells of remnant kidneys were suppressed due to elevated levels of TGF-β, tumor necrosis factor (TNF)-α, and oxidative stress. Besides, overexpression of KLF15 down-regulated the levels of fibronectin as well as type IV collagen mRNAs in HEK293 and mesangial cells, and *Klf15*-KO mice were found to develop glomerulosclerosis after uninephrectomy 45, 47. These findings indicate that KLF15 may exert antifibrotic roles in mesangial cells. Other studies with similar results have also confirmed this hypothesis 36.

Apart from mesangial cells, the expression of KLF15 in the renal interstitium of 5/6-nephrectomized rats was also explored, and a significant decrease in KLF15 levels was observed 17. I*n vitro* experiments demonstrated that KLF15 attenuated the deposition of extracellular matrix and CTGF by inhibiting TGF-β1-mediated Jun N-terminal kinase (JNK)/mitogen-activated protein kinase (MAPK) and ERK/MAPK signaling pathways. Therefore, KLF15 may play an antifibrotic role in the interstitium of remnant kidneys through the molecular mechanisms mentioned above 17.

It is known that the main pathological characteristics of hypertension-induced kidney injury include glomerulosclerosis, tubulointerstitial fibrosis, and proteinuria 28. A recent study suggested that the deacetylation of KLF15 by Sirtuin 3 (SIRT3) may decrease the expression of fibronectin and type IV collagen in immortalized mouse podocyte MPC-5 cells and protect against hypertensive nephropathy 48.

## Challenges

KLF15 was first identified in 2000 as a Kidney-enriched Krüppel-like factor, after which a great number of studies have focused on its physiological and pathological roles. Recent studies have greatly expanded our knowledge of its diverse functions in various human organs, tissues, and cells. In this review, we focused on KLF15 role in kidney biology and highlighted its role as a protective factor against podocyte injury, renal fibrosis, and other pathological processes. As the dysregulation of KLF15 has been shown to be implicated in the progression of CKD, KLF15 is expected to be a novel therapeutic target.

Remarkably, the podocyte-specific induction of *Klf15* exhibits a renal-protective role in proteinuric murine models 13. Future studies are warranted to examine whether a prolonged induction is safe and effective, and whether other cell-specific or a global induction will exhibit similar beneficial roles. In addition, what roles KLF15 may play in other glomerular or tubular diseases, and which pathway may be involved, also need to be investigated in multiple directions.

Present studies on the role of KLF15 in kidney biology mainly focus on podocyte injury, mesangial pathology, and renal fibrosis. In this review, we have introduced several major functions and the corresponding molecular mechanisms of KLF15, involving the restoration of podocyte differentiation markers, the inhibition of the pathological proliferation of mesangial cells, and the antifibrotic effect in mesangial cells and the tubulointerstitium. Different roles in different cells indicate a cell-specific role of KLF15 in kidneys. The functions and molecular mechanisms of KLF15 in other cells in the kidney need further investigation. Future research concerning the functions of KLF15 in kidney development will promote the understanding of this issue. In addition, since KLF15 regulates target gene expression by interacting with binding partners 25, it would be interesting to know if the function of KLF15 will be lost or changed once these protein-protein interactions are destroyed. Therefore, it will be a significant task to identify the specific protein binding partners and the downstream target of KLF15 in diverse cell types, thus enabling the development of potential therapeutic targets.

## Conclusions

In this review, we summarized the diverse roles of KLF15 and elaborated on its protective effects in kidney diseases (Table 1), providing new insights into the progression and therapy of CKD. KLF15 is expected to be a potential therapeutic target for glomerular diseases and renal fibrosis. Verifying specific functional mechanisms of KLF15 in different cell types or diseases will be a promising prospect in moving forward in precision medicine.

## Figures and Tables

**Table 1:** Functions of KLF15 in kidney biology

	Experimental model/cell	Functions and relevant mechanisms in kidney biology	Refs
**Tubular physiology**	Rat tubular cells.	KLF15 suppressed the expression of CLC-K1 and CLC-K2 in the thin descending limb of the loop of Henle and inner medullary collecting ducts by competing for promoter binding with MAZ, contributing to the kidney-specific expression of CLC-K1 and CLC-K2.	(23)
**Podocyte injury**	Podocyte-specific *Klf15*-KO mice treated with/without LPS or ADR; kidney biopsies from patients with FSGS and HIVAN.	KLF15 is a key regulator of podocyte differentiation and protects against podocyte injury via transcriptionally regulating podocyte-specific genes (*Synaptopodin, Podocin, and Nephrin*).	(27)
	Human podocytes; proteinuric murine models.	KLF15 is important in mediating the beneficial effects of GCs in podocytes. Loss of KLF15 led to a destabilization of the actin cytoskeleton and an increased podocyte injury, while overexpression of KLF15 stabilized the actin cytoskeleton under podocyte stress.	(16)
	Podocyte-specific induction of* Klf15* in ADR-induced and HIV-1 transgenic (Tg26) proteinuric murine models.	KLF15 is renal-protective. Podocyte-specific induction of *Klf15* attenuated renal fibrosis, inflammation and podocyte injury, and improved renal function. Mechanisms include activation of pathways implicated in the restoration of podocyte differentiation markers, the stabilization of the actin cytoskeleton, and focal adhesion.	(13)
**Mesangial pathology**	Classic anti-Thy1 mesangial proliferative nephritis rat model.	KLF15 expression in mesangial cells inhibited cell proliferation by down-regulating the expression of cell cycle regulation factor E2F1.	(37)
**Lupus Nephritis**	*In vivo* LN mouse model.	Combined treatment of Hydroxychloroquine and artemisinin exerted renal-protective effects by increasing the levels of KLF15, leading to a decreased expression levels of NF-κB, and consequently suppressing the inflammatory response.	(21)
**Renal fibrosis**	UUO mice (progressive renal fibrosis model); *Foxd1-Cre Klf15^fl/fl^* cells in UUO mice and Ang II-treated mice.	KLF15 has an antifibrotic effect in renal fibrosis by inhibiting the canonical Wnt/β-catenin pathway.	(46)
	Ang II-treated mice.	KLF15 inhibited the expression of Ang II-induced CTGF by suppressing the recruitment of the co-activator P/CAF to the *CTGF* promoter, exerting an early antifibrotic effect in renal fibrosis.	(18)
	5/6-nephrectomized rat; murine mesangial cells and HEK293 cells transfected with a mouse KLF15 complementary DNA expression vector.	The expression of KLF15 in mesangial cells was suppressed in the remnant kidney due to elevated levels of TGF-β, TNF-α, and oxidative stress. Overexpression of KLF15 in mesangial cells and HEK293 cells down-regulated the expression of fibronectin and type IV collagen mRNAs.	(45,47)
	5/6-nephrectomized rat.	KLF15 attenuated the deposition of extracellular matrix and CTGF by inhibiting TGF-β1-mediated JNK/MAPK and ERK/MAPK signaling pathways. Thus, KLF15 plays an antifibrotic role in renal interstitial fibrosis.	(17)
	MPC-5 cells.	KLF15 protects against hypertensive nephropathy by decreasing the expression levels of fibronectin and type IV collagen.	(48)

## Abbreviations

KLF15: Krüppel-like factor 15
KO: knockout
LPS: lipopolysaccharide
ADR: Adriamycin
FSGS: Focal Segmental Glomerulosclerosis
HIVAN: HIV-associated nephropathy
GCs: Glucocorticoids
LN: Lupus nephritis
NF-κB: nuclear factor-κB
MAZ: myc-associated zinc-finger
UUO: unilateral ureteric obstruction
Ang II: angiotensin II
CTGF: connective tissue growth factor
TGF-β: transforming growth factor-β
TNF-α: tumor necrosis factor-α
ERK: extracellular-regulated kinase
MAPK: mitogen-activated protein kinase
JNK: Jun N-terminal kinase
MPC-5 cells: conditionally immortalized mouse podocytes
P/CAF: p300/CREB-binding protein-associated factor
RA: retinoic acid
cAMP: cyclic adenosine monophosphate
PKA: protein kinase A
CRE: cAMP response element
CREB: cAMP response element-binding protein
WT1: Wilms Tumor 1
GR: glucocorticoids receptor
GRE: GC response element
CDK2: cyclin-dependent kinase 2
